# Sustainable human health: practical innovations of “Holding Groups for Mutual Support” (HGMS) in active aging

**DOI:** 10.3389/fpubh.2025.1558492

**Published:** 2025-04-23

**Authors:** Zhihong Zeng, Chaoran Wang, Shengnan Wu

**Affiliations:** ^1^School of Public Administration, Hangzhou Normal University, Hangzhou, China; ^2^School of Public Affairs, Zhejiang University, Hangzhou, China; ^3^Center of Emergency Management, Chongqing Economic and Social Development Research Institute, Chongqing, China; ^4^Center of Emergency Management, Chongqing Academy of Governance, Chongqing, China

**Keywords:** sustainable human health, active aging, age-friendly environment, older adults, holding groups for mutual support

## Abstract

In a rapidly aging global society, “sustainable human health” and “active aging” have become significant international concerns. The growing demand for high-quality older adult care, alongside resource disparities, challenges sustainable active aging strategies. As the nation with the largest older adult population, China faces the urgent need to establish a sustainable older adult care system that enhances the health and well-being of older adults. “Holding Groups for Mutual Support (HGMS)” promotes autonomous aging choices and emphasizes dignified living through “home, gathering, mutual support, and self-care,” addressing key issues like resource imbalances and inadequate policies. This study utilizes a mixed-methods approach that integrates both qualitative and quantitative techniques to systematically investigate the perspectives of 17 members aged 60 to 80 from the HGMS, alongside insights from 146 key stakeholders, which include family members, government representatives, enterprises, and mass media. This research identifies stakeholder attitudes toward the HGMS model, analyzes its challenges in the current social context, and explores its potential impacts on sustainable older adult care. Findings indicate that the HGMS model improves resource sharing and environmental protection while addressing the supply-demand imbalance in traditional older adult care. However, challenges remain, including inadequate policy backing, cognitive biases, and uneven resource allocation. This study provides valuable insights for developing sustainable older adult care systems in China and globally.

## Introduction

Human health constitutes a pivotal aspect of the Sustainable Development Goals (SDGs), which encompass essential elements including political democracy, economic prosperity, cultural inclusion, social equity, and ecological sustainability. As the quality of human life improves and medical technology advances continuously, there has been a notable increase in the global average life expectancy. According to United Nations projections, the global population’s annual growth rate is expected to decrease to around 0.5% by 2050, the average life expectancy worldwide continues to rise, climbing from 63.8 years in 1990 to 77.3 years in 2024, with a projected increase to 77.4 years by 2054. For the group of countries with populations that have already peaked, it is anticipated that individuals aged 65 and older will grow rapidly, nearly doubling between 2024 and 2054 from 17 to 33% (1). However, the increasing trend of global aging, in conjunction with the high incidence of chronic diseases among the older adults, poses a significant threat not only to the physical health of this demographic but also results in considerable economic burdens. Furthermore, this situation presents substantial challenges to socioeconomic systems, healthcare infrastructures, and social security frameworks, ultimately affecting overall social welfare (2). Therefore, the transition of older adults from a passive acceptance of treatment to an active engagement in health maintenance is of paramount importance. Traditionally, aging has been perceived as a process of decline, characterized by a deterioration in physical function, diminished social participation, declining mental health, and limited social welfare (3, 4). Recently, the concept of “active aging” has progressively altered the negative perceptions, showcasing the potential for older individuals to live vibrant and engaged lives. It portrays aging as a vibrant and positive journey, emphasizing the importance of enabling older individuals to lead healthy lives and actively contribute to society (5). The connotations primarily encompass three key aspects. Firstly, older individuals should be recognized not as burdens but as valuable assets to their communities and society at large. Secondly, it is essential for older adults to actively and healthily engage in the advancement of society (6). Lastly, it is the duty of both the government and society to take proactive measures to uphold the rights of older individuals to maintain their health and actively participate in civic affairs. In essence, active aging signifies a paradigm shift in human health that is consistent with the United Nations Sustainable Development Goals (SDGs) pertaining to equality, health, and well-being (7). This concept embodies a more holistic and individualized developmental philosophy, promoting the establishment of a more inclusive and mutually supportive society. Firstly, it moves away from the traditional emphasis solely on physical health towards a more holistic approach that considers the overall health of society. This shift underscores the crucial role of the government in leading and ensuring accountability in health governance. Secondly, the focus has broadened from solely the older adult population to encompass all members of society, examining the aging process of individuals from a health standpoint. This broader perspective highlights the importance of intergenerational social cohesion in response to aging population. In China, traditional family caregiving and institutional care are facing a gradual decline due to factors such as shrinking family sizes, rising living costs for the older adult, and fragile social support systems. These challenges are particularly acute in rural areas, where providing long-term care for the older adult has become a critical issue, underscoring the urgent need for innovative care models (8). Inspired by positive values like “mutual support in times of need, helping one another during illness, and fostering community care,” mutual aid pension models have begun to emerge, tailored to local contexts. Notable examples include rural happiness centers with mutual aid groups (9), mutual aid social organizations, and time banks for older adult care (10). Additionally, the emergence of the HGMS older people care model has been identified as a viable option within the realm of active aging, offering a solution to practical challenges in older adult (11).

HGMS originated from the co-housing older adult care model in Denmark during the 1960s and 1970s (12). Danish cohousing emphasized communal living, shared facilities, and resident-led decision-making, aiming to counteract social isolation through collective autonomy (13). This model inspired global variations (14), including Japan’s senior cooperatives, which emerged in the 1990s as member-driven organizations providing mutual aid services such as home-sharing, health advocacy, and cultural activities (15). While both Danish cohousing and Japanese cooperatives emphasize older adults’ self-determination, their governance models diverge: Danish communities frequently leverage public-private partnerships to support infrastructure development (14), whereas Japanese cooperatives rely primarily on grassroots mobilization with minimal governmental involvement (16). Drawing on these international precedents, the HGMS model integrates the principles of mutual assistance and communal living while tailoring its approach to China’s distinct sociocultural context. Initially, the model aimed to foster mutual assistance and communal living among older individuals. As the cohousing concept evolved, older people began forming their own communities to enhance their quality of life in their later years.

The definitions of the HGMS model vary widely, with different interpretations placing emphasis on various aspects. Some focus on the foundational principles and essential characteristics of participants (17), while others take a more functional approach (18). Recent research has highlighted that the five main dimensions appear essential for “ageing well at non-traditional home”: the health, affection, social contact, building and environment. Research indicates the existence of diverse forms of the HGMS, with three predominant approaches that have seen significant development in Chinese society: mutual aid in rural areas, self-help in urban settings, and community-based semi-assistance. This diversification responds to systemic gaps in China’s transitional care system, which struggles to address heterogeneous older adult needs ranging from mobility limitations in remote areas to ageism-driven social exclusion.

Addressing the unmet care and support needs of the aging population, as well as designing services and solutions that align with the needs and preferences of older adults, has emerged as an urgent public health priority (19). Within the context of China’s swift transition to an aging society, the current eldercare service system confronts two primary challenges. On one hand, the transitional care system inadequately addresses the complex demands of the older adult regarding quality eldercare. On the other hand, existing healthcare services are ill-prepared to accommodate the individual circumstances of older adults, such as limited income or insurance, reduced mobility or disability, rural or remote location, and negative self-perceptions of ageing (20). Thus, there is a pressing need for comprehensive older adult care services to effectively meet the needs of a substantial and growing older adult population (21). Conventional community-based (22), and institutional-based (23), are struggling to meet the evolving demands efficiently. In this context, the HGMS model emerges as a promising alternative, with the objective of advancing the aspiration for sustainable health and well-being within the framework of active aging.

## Materials and methods

### Concept of the holding groups for mutual support (HGMS)

HGMS was initiated in 2017 by two senior residents of Longevity Village in Pingyao Town, Hangzhou City, Zhejiang Province, China. Covering an area of 6.75 square kilometers and having a population of approximately 4,050. Longevity Village boasts an advantageous geographical location, accessible transportation, and pleasant living environment, making it an attractive place for retirees. Moreover, the village is close to the Liangzhu cultural site, an ancient civilization of considerable historical significance, dating back approximately 5,250 to 4,150 years. Despite these advantages, the village faces challenges. Firstly, the shortage of age-appropriate facilities has rendered their daily lives inconvenient, directly impacting the overall quality of life and social interactions. Secondly, the limited availability of diverse social activities and platforms has hindered opportunities for residents to forge new friendships. Lastly, many retirees experience feelings of aimlessness and a lack of achievement, compounded by the daunting task of selecting a suitable retirement community. They often face a dilemma between the high costs associated with quality facilities and the worries linked to lower-quality alternatives, all of which intensify their mental strain. Recognizing the loneliness of many older adult couples in the village, they decided to create a shared retirement living space by inviting peers from the surrounding area, thereby laying the groundwork for the HGMS model.

**Figure 1:**
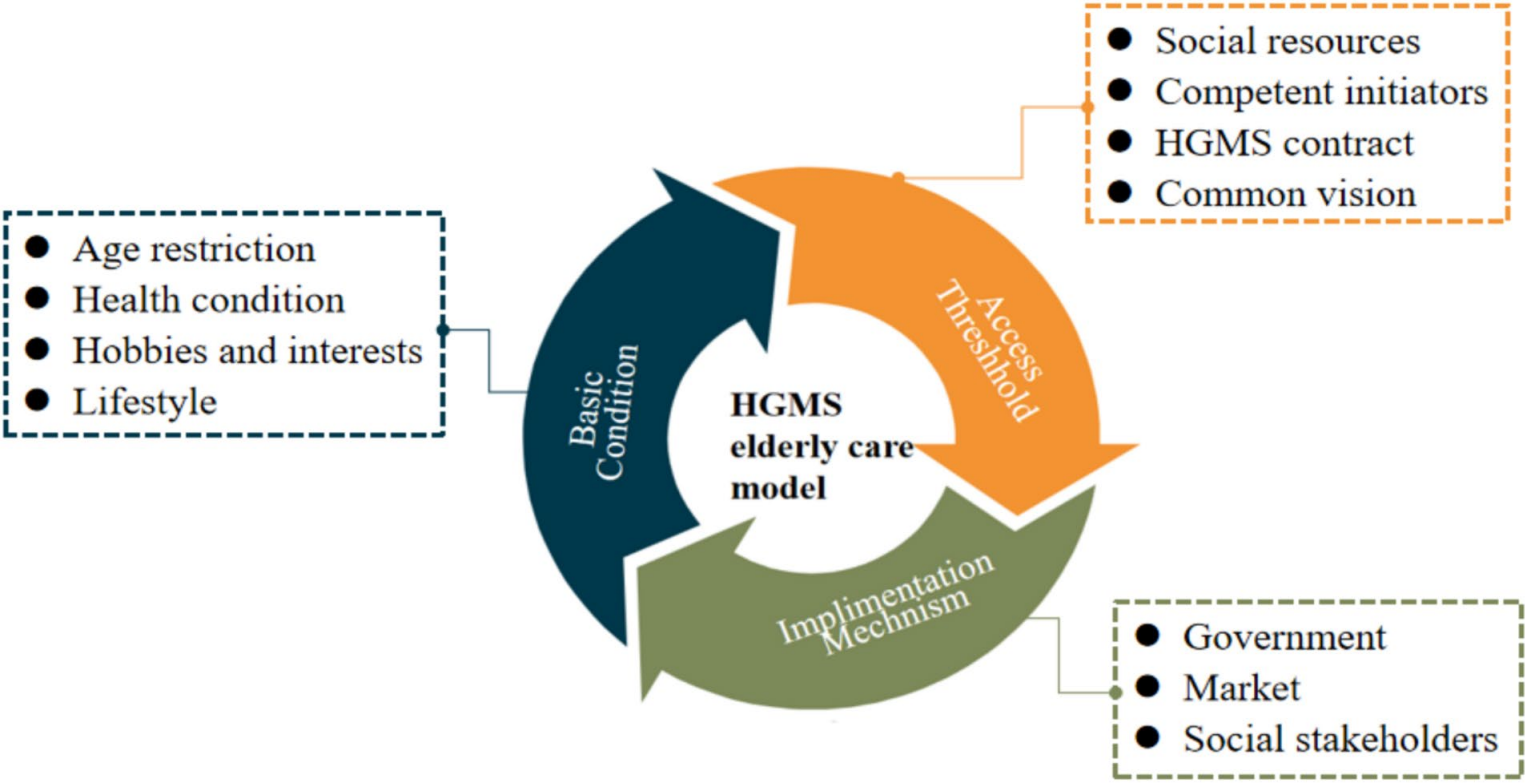
Concept of the Holding Groups for Mutual Support (HGMS).

Through extensive long-term observations and case studies, this paper articulates the core essence of the HGMS model and elaborates on its essential prerequisites and entry criteria. The HGMS model is defined as follows: “The HGMS model is a pioneering care model initiated by individuals aged 60 to 80 years who are healthy and capable of self-care. It is founded on common interests and lifestyle similarities. By incorporating voluntary agreements and the pooling of resources, it fosters a sustainable network for the provision of older adult care.” It is imperative to emphasize that the specified age range for HGMS members is flexible and may be adjusted in response to changes in national retirement policies (24). Variations in age do not hinder the effective implementation of the HGMS model. This model signifies a fresh perspective on older adult care, allowing older individuals to live together by mutually consenting to a care service contract to achieve consensus, as illustrated in Figure 1. HGMS functions as a self-governing care model that integrates underutilized societal resources, fulfills older adults’ inherent desire for dignified care, and alleviates the burden of older adult care on government services.

The HGMS model necessitates specific foundational elements, including a robust elder care infrastructure, capable group initiators, a mutually agreed-upon contract, and a common vision. These components are crucial for ensuring the sustainable functioning of the HGMS communal residence.

- Diverse social resources: Serving as the physical bedrock of the HGMS model, this facet demands ample residential space, convenient living amenities, and facilities that align with national elder care standards. Moreover, specialized equipment, particularly for emergencies like distress calls and rescue operations, is essential. These amenities can either be owned by the HGMS initiator or provided by third parties, with costs shared among members.
- Competent HGMS initiators: The success of the HGMS model hinges on these key individuals. They must possess a clear purpose, enthusiasm, resilience, adept management and coordination skills, and the capacity to take on managerial responsibilities within the HGMS framework.
- HGMS cohabitation agreement: Serving as the behavioral guideline for the HGMS model, this contract is voluntarily signed by the older adult residents. It outlines the rights and responsibilities of members during their shared occupancy and establishes common principles for communal living, including cost-sharing for daily expenses, duty assignments, property protection, and cohabitation rules. Typically, older individuals rely on this agreement to negotiate official matters and resolve disputes, fostering a foundational consensus during their shared residency. The contract stipulates those initiators hold democratic meetings to address challenges.
- A common vision for the HGMS communal residence: This aspect embodies the spiritual essence of HGMS. Older individuals exhibit a strong desire to come unite around a common vision—to enjoy a fulfilling and engaging retirement experience through the HGMS model. Throughout this process, these individuals cultivate a sense of unity and warmth within their shared household.

Beyond the fundamental prerequisites of the HGMS model, there are specific entry criteria for prospective members, encompassing age, health status, interests, and lifestyle compatibility.

Age criteria: Prospective members should fall within the age bracket of 60 to 80 years old. Individuals within this age range are typically physically active, relatively unencumbered by familial responsibilities, and possess the freedom and motivation to enhance their quality of life in their later years.
Health status: Prospective members should maintain good physical and mental health, self-sufficient in daily activities and willing to contribute to the communal chores of the group household, thereby avoiding imposing undue burdens on fellow members.
Interests and hobbies: Prospective members should share common hobbies and interests that enrich leisure time within the community, fostering enduring bonds among residents.
Lifestyle compatibility: Prospective members should exhibit similar living habits, demonstrating the capacity to accommodate and adapt to one another in terms of language, dietary preferences, living arrangements, customs, and more. This mutual compatibility is essential for fostering a harmonious and joyful living environment for the older adult residents within the HGMS model.

### Development of the holding groups for mutual support

The development of HGMS has been a multifaceted journey that can be broadly categorized into four distinct stages. The study will concentrate on the preparation and implementation phases, as they encapsulate the foundational principles of HGMS. The subsequent discussion will provide a detailed explanation of these two phases, revealing critical insights into the core components that characterize the HGMS. In contrast, the stagnation and reinvigoration phases underscore the various risks and challenges faced by the initiator, offering valuable lessons learned throughout the process.

**Figure 2:**
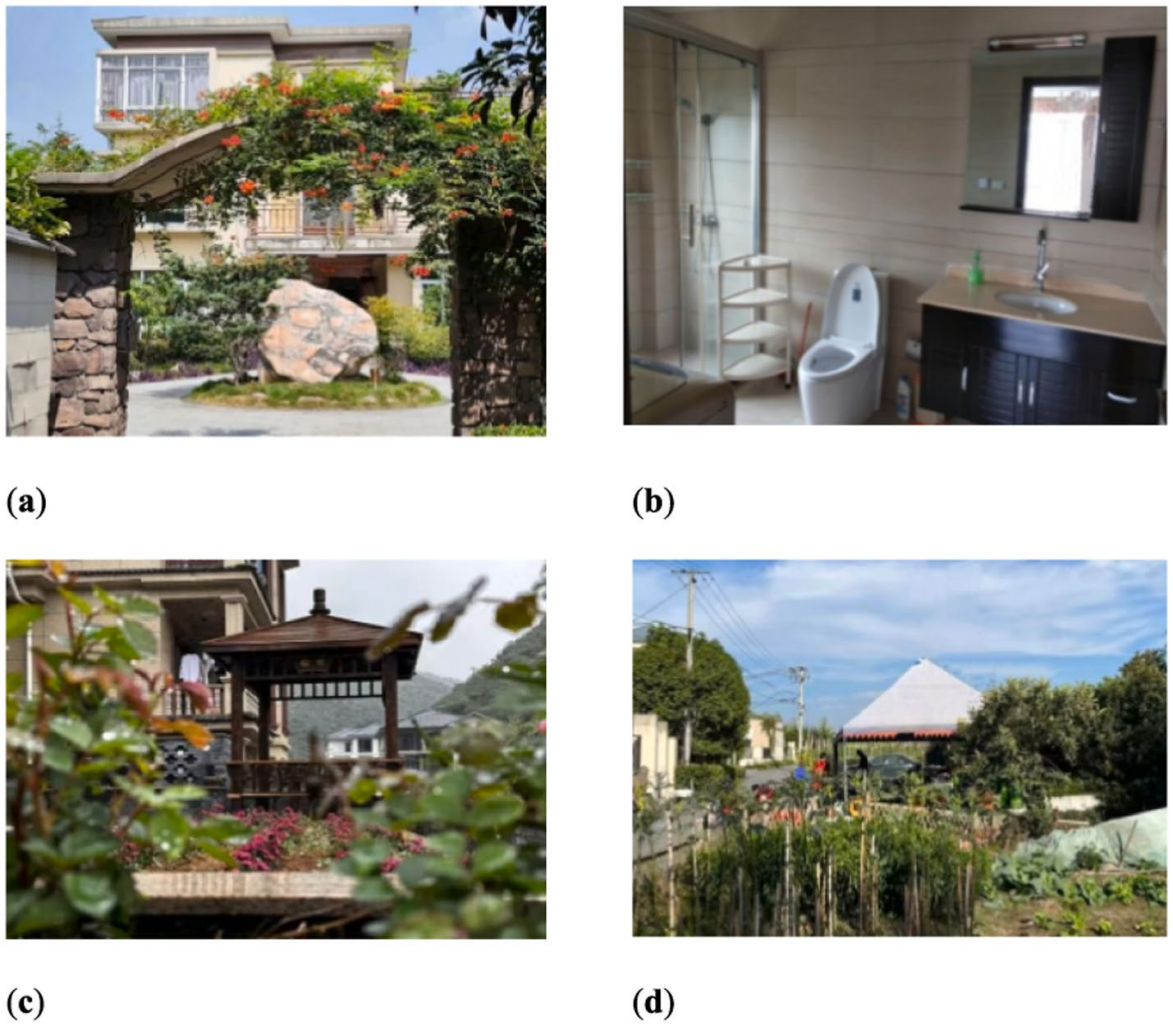
Age-friendly transformation of the HGMS in the Longevity Village. **(a)** Overlook of the shared house; **(b)** Redecorated bathrooms with aged-friendly facilities; **(c)** View of the garden; **(d)** Scene of the half-acre vegetable garden.

- Preparation Phase (September 2016–May 2017): (1) Upgrade and transform infrastructure for age-friendliness (see Figure 2). (2) Gather practical insights and effective practices for operation. (3) Recruit the HGMS members.
- Implementation Phase (July 2017–December 2019): (1) Develop and formalize a cohabitation agreement. (2) Adapt and achieve a collective consensus among members. (3) Improve the HGMS model in spiral continuously.
- Stagnation Phase (January 2020–January 2023): (1) Stagnate and transit to home care due to the covid-19 pandemic. (2) Construct a closely-knit online community.
- Reactivation Phase (February 2023—Present): (1) Renew the desire to reactivate the HGMS model. (2) Begin strategizing the reinstatement of HGMS operations. (3) Confront with a variety of unforeseen obstacles.

It is important to highlight that HGMS is committed to providing each member with high-quality care, a comfortable living environment, all at an affordable cost of approximately 1,500 RMB per person per month.

### Study design and setting

A participatory tracking survey and semi-structured interviews were conducted with older residents living in the Longevity Village HGMS model, alongside the distribution of random questionnaires to stakeholders involved. The study assessed older adults’ perceptions regarding the HGMS model. Additionally, it explored the connotations of the HGMS model, membership criteria, and the roles of stakeholders. The research also analyzed the advantages and disadvantages of the HGMS model and proposed optimization strategies. The duration of the study was from October 2017 to December 2024. Given the potential impact of the interview method on participants’ natural responses, the discomfort of some older adult participants with written documents, and the necessity to protect participants’ privacy, this study employed an oral consent procedure to obtain informed consent from all participants prior to conducting interviews and case studies. Detailed documentation of this process is included in the participant records. This procedure aligns with the Declaration of Helsinki and has been approved from the University Research and Ethics Committee (2017PA008).

### Participants

This study focused on the HGMS model within Longevity Village, incorporating insights from a diverse range of stakeholders. Participants were recruited through direct invitations, social media, and online platforms between October 10th, 2017, and December 30th, 2024. Participants included HGMS initiators, members, their families, government officials, business representatives, and social media platforms. A total of 17 older individuals participated in the HGMS model, consisting of 2 initiators and 15 members. It is noteworthy that the HGMS has experienced some personnel turnover. The initiators primarily invited 11 older adult participants from over 100 applications, but some older adult individuals later withdrew due to family or health reasons, while others joined the program. The sample of older individuals interviewed displayed a balanced gender representation, with 47.1% males and 52.9% females, and an age range of 60 to 80 years. The selected HGMS participants possessed high educational and professional backgrounds, including workers, teachers, doctors, and engineers. In-depth retrospective interviews were conducted to understand entry and exit dynamics within the community. Demographic characteristics of survey respondents are reported in Table 1.

**Table 1:** Demographic characteristics of survey respondents (*N* = 17).

Characteristic		*n* (%)
Gender	Male	8 (47.1%)
	Female	9 (52.9%)
Age	60–65	6 (35.2%)
	66–70	8 (47.1%)
	71–75	2 (11.8%)
	76–80	1 (5.9%)
Marital status	Married	16 (94.1%)
	Widowed	1 (5.8%)
Education	Elementary education	3 (17.6%)
	Secondary education	7 (41.1%)
	Junior college education	2 (11.8%)
	Bachelor’s degree	3 (17.6%)
	Other	2 (11.8%)
Pre-retirement occupation	Worker	5 (29.4%)
	Teacher	1 (5.8%)
	Doctor	2 (11.8%)
	Engineer	1 (5.8%)
	Private sector employee	2 (11.8%)
	Individual entrepreneur	2 (11.8%)
	Other	4 (23.5%)

*All data in this table are from a survey conducted with the HGMS participants.

### Data collection

This study adopted a participatory observation methodology, where members of the research team actively participated in the development of the HGMS model and served as caregivers for the older adult. From October 2017 to December 2024, a wide range of perspectives were continuously monitored, encompassing the feelings of older adults, media reports, and social feedback. Focused interviews commenced in October 2017, involving 17 initial participants, especially the two primary initiators. These interviews probed into the needs and goals of care services, attitudes towards the HGMS model, and identified challenges as well as strategies for addressing them. Based on participant feedback, the study refined the questionnaire structure and interview outlines, initiating a pilot test with eight local older adult individuals to fine-tune specific questions. A follow-up survey was launched in March 2018 to collect insights on the perspectives and expectations of older individuals through questionnaires and semi-structured interviews at strategic locations in Hangzhou, each lasting at least 30 min. Additionally, during the stagnation phase of the HGMS, the research team pivoted from participatory surveys to online tracking methods (including telephone and video interviews) to ensure continued engagement with older adults.

### Data analysis

Data analysis was conducted using SPSS 26.0 software to evaluate the attitudes and of older adults aged 60–80 towards the HGMS model and their roles within such residential environment. First, descriptive statistics were calculated to summarize participants’ demographic characteristics, including gender, age, marital status, pre-retirement occupation, and education level. Second, inferential statistical analyses identified significant differences in attitudes based on roles variables. Furthermore, qualitative data from open-ended survey responses were thematically analysed to complement quantitative findings, providing deeper insights into older individuals’ perceptions and lived experiences within HGMS.

## Results

Based on Likert’s five-point scale (strongly agree, agree, not necessarily, disagree, strongly disagree), this study preliminarily assessed the basic attitudes of older adults, categorizing them into three classifications: support, neutrality, and opposition. An analysis of stakeholder attitudes toward the “Holding Groups for Mutual Support” (HGMS) revealed significant variations among different groups. As illustrated in Table 2, the total number of respondents fluctuated across categories, with an overall participation of 218 individuals. Among the two initiators of the HGMS, all expressed support (100%), with no neutral or opposing views. Similarly, the 15 HGMS members showed no neutral or opposing responses. Among the 45 willing participants, 32 (71.1%) supported HGMS, while 13 (28.9%) were neutral, with no opponents. In the lager random group of 156, 49 (31.4%) supported, 91 (58.3%) were neutral, and 16 (10.3%) opposed HGMS, reflecting a wide range of perspectives.

**Table 2:** Basic attitudes of respondents (*N* = 218).

Respondents^a^		*n* (%)
HGMS Initiator (*N* = 2)	Supporters	2 (100%)
HGMS members (*N* = 15)	Supporters	15 (100%)
HGMS ready-to participant (*N* = 45)	Supporters	32 (71.1%)
	Neutral	13 (28.9%)
Random respondents (*N* = 156)	Supporters	49 (31.4%)
	Neutral	91 (58.3%)
	Opponents	16 (10.3%)

a Respondents are the older adults aged 60 to 80.

Through the integration of quantitative and qualitative results, three key themes emerged, shedding light on the complexities of adopting Holding Groups for Mutual Support (HGMS): (1) Diverse Perspectives on HGMS Among Stakeholder Groups; (2) Role Differentiation and Collaborative Dynamics; (3) Neutrality and Opposition: Barriers to Adoption.

### Theme 1: diverse perspectives on HGMS among stakeholder groups

The analysis uncovered notable differences in stakeholders’ attitudes toward the Holding Groups for Mutual Support (HGMS). Using a Likert 5-point scale, participants fell into three categories: support, neutrality, and opposition. These divergent attitudes highlight varying levels of engagement and acceptance, shaped by stakeholders’ unique experiences and expectations regarding community-based older adult care initiatives. As one 72-year-old participant (H1) expressed:

I like the concept of support groups, but I need to see how they function in real-life situations before I can fully commit to them.

This sentiment emphasizes the importance of practical implementation and transparent operations in building trust among stakeholders.

### Theme 2: role differentiation and collaborative dynamics

The study highlighted clear role distinctions among stakeholders within the HGMS framework, underscoring the critical role of collaboration for successful implementation. HGMS initiators took on key responsibilities, including integrating members, building operational infrastructure, establishing guiding principles, and managing daily operations. Meanwhile, HGMS members and their families played an essential part by managing their own day-to-day activities while actively engaging in program initiatives.

As one family member shared:

It’s a shared responsibility to make sure everyone is doing well… we need to stay involved and informed.

This insight emphasizes the collective effort and teamwork necessary to foster a supportive and effective community-centered care environment.

### Theme 3: neutrality and opposition: barriers to adoption

While many participants expressed support for HGMS, the prevalence of neutral and opposing attitudes reveals potential challenges to broader community adoption. In particular, over half of a larger randomized group reported a neutral stance, signaling hesitancy that could stall acceptance of the model. Understanding the underlying factors driving these reservations is essential for overcoming barriers to engagement. One participant summed up this cautious perspective:

I believe mutual support is important, but I need more confidence in how it will be organized and sustained before I can fully trust it.

Meanwhile, the 10.3% of participants who opposed the model voiced concerns not only about its operational effectiveness but also about the reliability of support from various stakeholders. This mixed feedback underscores the need for focused outreach efforts to transform neutrality and opposition into active support, fostering greater inclusivity and building trust within the community.

### Main roles and attitudes of HGMS stakeholders

This study identified the roles and attitude variations of stakeholders in the HGMS through extensive long-term observations and interviews, along with Likert scale data gathered from main stakeholders (see Table 3). In terms of roles, HGMS initiators were entrusted with integrating organizational members, establishing requisite infrastructure, formulating fundamental standards, and supervising daily operations. HGMS members and their families were accountable for managing personal daily lives and participating in various project activities, while family members were obligated to provide regular visits and support. Government agencies supervised daily operations and offered policy support to guarantee compliance and effectiveness. Enterprises focused on providing high-quality elder care products and services, thereby promoting the aging industry. The mass media played a role in monitoring progress and raising societal awareness. The interactions and collaborations among these stakeholders were essential for the sustainability and societal acceptance of the HGMS model.

**Table 3:** Perception of stakeholders towards the HGMS (*N* = 146).

Stakeholders	Perception	*n* (%)
HGMS members (*N* = 17)	Felt satisfied with the HGMS	15 (88.2%)
	Felt my life quality improved	16 (94.1%)
	Felt necessary to establish cohabitation agreements	17 (100%)
	Felt positive impact on my social interactions and mental health	16 (94.1%)
	Felt potential risks and hazards associated with HGMS	17 (100%)
Families of the older adult (*N* = 28)	Felt supportive of HGMS development	25 (89.3%)
	Felt life quality of the older adult improved	26 (92.9%)
	Felt positive impact on older adult social interactions and mental health	24 (85.7%)
	Felt potential risks and hazards associated with HGMS	28 (100%)
	Felt emotional communication between older adult and family fostered	23 (82.1%)
Government (N = 16)	Felt supportive of HGMS development	10 (62.5%)
	Felt beneficial for promoting active aging	13 (81.3%)
	Felt potential risks and hazards associated with HGMS	15 (93.8%)
	Felt necessary to cultivate public policy for HGMS	12 (75%)
	Felt responsible for guiding the community participate in HGMS	16 (100%)
Enterprises (*N* = 35)	Felt supportive of HGMS development	15 (42.9%)
	Felt beneficial for promoting active aging	25 (71.4%)
	Felt potential risks and hazards associated with HGMS	30 (85.7%)
	Felt HGMS offered new opportunities in insurance and business	28 (80%)
	Felt corporate social responsibility reflected in active aging	20 (57.1%)
Mass media (*N* = 50)	Felt supportive of HGMS development	40 (80%)
	Felt beneficial for promoting active aging	42 (84%)
	Felt potential risks and hazards associated with HGMS	48 (96%)
	Felt widespread dissemination of HGMS promoted	32 (64%)
	Felt responsible for guiding the older adult to join HGMS	30 (60%)

^aStrongly agree or agree. bThe sample consisted of 2 initiators and 15 members of HGMS.^

**Figure 3:**
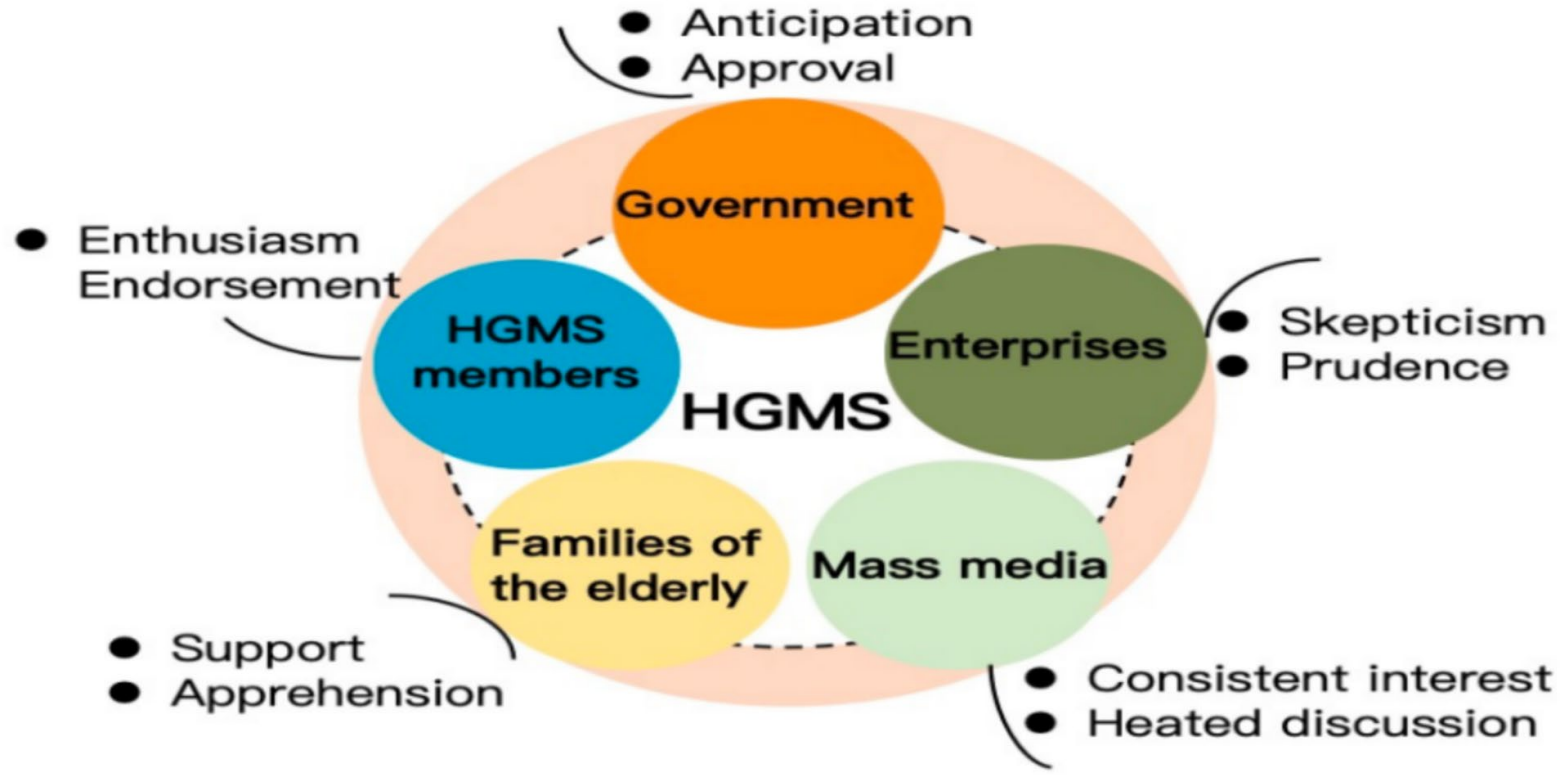
The attitude of stakeholders towards the HGMS model.

Regarding attitudes, survey results presented in Figure 3 reveal distinct perspectives among various stakeholders toward the HGMS. In general, stakeholders expressed overall support for the HGMS model, although enterprises tended to display skepticism and prudence. Feedback from the Likert scale (see Table 3.) indicated that HGMS members and their families largely perceived the model as beneficial for improving the quality of life among the older adult and positively affecting social interactions and mental health. Notably, many participants emphasized the importance of establishing cohabitation agreements to strengthen support systems. While numerous stakeholders expressed support for HGMS development and recognized its advantages for active aging, concerns about potential risks and challenges were also acknowledged. This underscored the necessity for ongoing public policy cultivation and community engagement to ensure the effectiveness and sustainability of the model, fostering emotional connections between the older adult and their families.

Furthermore, research indicated a correlation between the attitudes of HGMS stakeholders and their roles (see Table 4). This study categorized stakeholders’ attitudes into four dimensions: willingness, satisfaction, attention, and expectation. The research findings indicated that all correlations were statistically significant (*p* < 0.01), demonstrating a meaningful relationship between stakeholders’ role within HGMS and the various issues examined.

**Table 4:** The correlation between the attitudes and roles of HGMS stakeholders.

Variable	M	SD	Role	Willingness	Satisfaction	Attention
Role	3.5	1.425	1			
Willingness	2.32	0.908	0.442^**^	1		
Satisfaction	2.22	0.747	0.330^**^	0.762^**^	1	
Attention	2.03	0.626	0.475^**^	0.636^**^	0.588^**^	1
Expectation	2.27	0.649	0.403^**^	0.660^**^	0.686^**^	0.452^**^

** The correlation is significant at the 0.01 level.

### Obstacles to HGMS

As part of this study, subsequent investigations identified several barriers that impede the sustainable development of the longevity village HGMS model, which were categorized into four main aspects. Firstly, the inadequate management and operational mechanisms, particularly the absence of a formal access and withdrawal system, posed challenges for HGMS members’ interactions with older individuals who temporarily withdrew from the community. Secondly, the limited care resources and supporting facilities led to a sense of monotony among older residents after prolonged periods of living together. Thirdly, the HGMS model lacked the support of favorable policies for an extended period, resulting in rising living costs for members and a decline in the quality of care services provided. Lastly, the model of elder care within HGMS lacked external resources for assistance, leaving older residents without social support and professional guidance. Subsequent sections of this paper would delve into each of these obstacles in detail.

- Absence of access and withdrawal mechanism: The lack of a formal access and withdrawal mechanism resulted in frequent turnover among HGMS members, creating challenges for both the initiators and the older adult residents. This constant flux not only increased the workload for the initiator in recruiting new members but also led to instability, as older individuals had to continually adapt to new neighbors. The reasons for members’ departures varied widely, from sudden parental illnesses to medical emergencies and accidents. This absence of a structured access and withdrawal process highlighted the unsustainability of relying solely on sponsor-driven recruitment. Establishing a clear mechanism for access and withdrawal is crucial for maintaining the stability and effectiveness of the HGMS model in the long term.
- Insufficient infrastructure resulting from constrained resource: Insufficient infrastructure resulting from limited financial resources posed challenges for the HGMS initiator in providing facilities and resources comparable to those of professional care institutions. The limited budget made it challenging to match national safety standards and medical protocols required for care facilities in China. Additionally, the lack of diverse entertainment options further exacerbated the issue. Unlike professional care facilities in China that offer a range of amenities such as chess and card rooms, study areas, art studios, and game rooms, older residents in HGMS often found themselves bored with the limited recreational options available. This highlighted the urgent need for enhanced recreational facilities within the HGMS model.
- Lack of preferential policies: Although the HGMS model had garnered recognition from the government and other stakeholders as an innovative approach, the absence of concrete supportive measures had resulted in a significant lack of favorable policies. Traditional elder care facilities benefit from discounted living expenses for amenities such as telephone, satellite TV, and internet under a tiered pricing structure, while HGMS currently faces per capita costs that are significantly higher than those of average core families or elder care institutions. This disparity created an unfavorable situation for the older adult within HGMS, leading to dissatisfaction among both initiators and members.
- Shortage of external assistance: The lack of external support and assistance represented a significant challenge within the HGMS model. Despite extensive media coverage and global attention, initiators often felt overwhelmed and frustrated by the magnitude of their responsibilities, facing both psychological burdens and practical challenges in seeking external assistance to navigate unknown risks. Disputes among HGMS members over rights and responsibilities complicated mediation efforts for initiators, while a lack of experience in organizing recreational activities and group events added to their difficulties. Originally, initiators envisioned creating a “happy group of mutual support,” but they found themselves inadvertently transformed into “servants” tasked with managing chaos among members. This fundamental role reversal left the initiators feeling overwhelmed. Additionally, gaps knowledge regarding legal matters, insurance, and emergency response left both initiators and members struggling in unfamiliar territory, making it impractical to expect them to acquire such specialized skills. These findings highlighted the limitations of relying solely on internal resources within the shared living environment to address unforeseen risks and challenges, emphasizing the need for external support to bolster the resilience of the HGMS model. In practice, external resources were not leveraged effectively, necessitating the incorporation of external forces to complement internal strengths.

## Discussion

This study evaluated the HGMS model from the perspectives of active aging and sustainable health, offering in-depth insights into its operational modalities and processes, the roles and attitudes of stakeholders, along with its advantages and disadvantages. To the best of our knowledge, the HGMS model is the first instance of a “group pension” spontaneously initiated by the older adult themselves in China. Unlike previous research on community-based elder care, this study found that the HGMS model relies on direct involvement from its initiators to match participants and operates autonomously based on member cooperation. However, these unique characteristics introduce challenges, such as a singular management mechanism, insufficient policy backing, and limited social engagement.

Overall, these findings indicate that HGMS is not merely a housing solution, they embody a progressive social strategy for older adult care aimed at enhancing the quality of life for seniors with limited resources. The primary focus of this model is not solely to meet the material needs of the older adult, but rather to prioritize daily care and mental well-being, thereby fostering a positive environment through communal living (25). The HGMS model is well-suited for retirees who prioritize social inclusion, physical and mental health, and tangible support. Under the “9,073” pattern of China’s pension system (26), while the implementation of the HGMS model is not universally accessible, it possesses the potential to address the disparity between the supply and demand for traditional older adult care options (27). On one hand, HGMS effectively encourages social interaction among older adults through various shared activities. Residents participate in collective tasks such as shopping, meal preparation, and conflict resolution, as well as recreational activities like chess, card games, exercise, conversation, and travel. These interactions help reduce feelings of loneliness, strengthen social connections, and enhance emotional fulfilment. Moreover, the HGMS model enables seniors to continue contributing to society post-retirement, reframing their role from mere recipients of care to active participants. By actively involving older adults in community and volunteer activities, the model encourages their participation in political and social affairs, thereby upholding their dignity and value (28, 29). On the other hand, the HGMS model has great potential in alleviating the financial strains for older individuals. Members reduce shared daily expenses through resource sharing and provide mutual support, alleviating the care-giving burden on their families. This approach provides older adults with financial stability and a sense of security, while also mitigating the crisis associated with the loss of professional identity that often accompanies retirement (30). Furthermore, from the perspectives of architecture and environmental sustainability, HGMS underscores the significance of shared spatial design while prioritizing the construction of barrier-free facilities and eco-friendly environments. This approach not only enhances the comfort of older adult residents and minimizes resource consumption linked to redundant infrastructure development (31) but also aligns with the principles of contemporary sustainable development. Consequently, it offers valuable references and insights for the future advancement of older adult care communities.

Although HGMS has demonstrated positive outcomes in practice, it still encounters various challenges in its pursuit of sustainable development. From the perspectives of key stakeholders, infrastructure, and external support, the model encounters issues related to feasibility, scalability, and sustainability. Firstly, concerning stakeholders, the HGMS model differs from shared housing based on familiar social circles by bringing a group of strangers together (32). As a temporary solution for elder care, HGMS has limitations in terms of member types, scale, and structure. As members age, their physical capabilities gradually decline, impacting their ability to remain self-sufficiency over time. This trend diminishes the model’s effectiveness. Moreover, the HGMS model has a limited target audience, with a maximum capacity of approximately 20 individuals, which restricts comprehensive emotional support. Furthermore, differences in personality, habits, language, and behavior among members may also lead to escalating conflicts, which may escalate if not addressed properly. Secondly, with regard to infrastructure, the medical and recreational facilities and services provided by HGMS are insufficient and fail to meet the standards of community or institutional elder care. Surveys reveal that daily medical services are inadequate, particularly in emergency situations. Members’ access to emergency assistance during sudden health crises is severely limited due to the absence of professional medical facilities, posing significant health risks. Finally, the external support available for HGMS appears to be quite restricted. Since its inception, HGMS has operated as an independent entity, with members collectively facing life’s challenges and forming strong emotional bonds. However, while this closeness fosters intimacy, it also isolates members from external support, as they often lack awareness and initiative in seeking assistance, which complicates access to professional support during difficulties.

Social innovation practices are instrumental at multiple levels in the formulation of future social development policies and principles, which are essential for sustainable community development (33). This study proposes an implementation mechanism designed to facilitate the sustainable development of the HGMS model. First, it is essential to strengthen the functionality of various mechanisms to ensure the sustainable development. This involves streamlining admission and exit procedures to effectively manage member turnover. HGMS initiators should collaborate with third-party organizations to maintain a comprehensive database of potential members, utilizing big data technology to identify suitable candidates promptly. Cohabitation agreements should require members to provide notice before leaving, allowing for quick filling of vacancies. Secondly, establishing a strong communication and coordination system is vital for resolving conflicts arising from diverse personalities and lifestyles. HGMS should implement effective internal communication tools, like a suggestion box, allowing members to express their opinions either openly or anonymously, with initiators actively responding to feedback. Additionally, a rotating “mediator” system can foster collective responsibility and encourage different perspectives in conflict resolution, promoting an atmosphere of respect and tolerance within the community. Given that the HGMS model’s core objective is to provide emotional support and a fulfilling life, establishing a robust democratic decision-making mechanism is equally crucial. Democratic meetings can effectively address issues related to cost-sharing, cooking preferences, recreational activities, task allocation, and conflict resolution.

Moreover, the government needs to create supportive policies that promote sustainable elder care models. The state plays a critical role in developing legislation that includes income support, healthcare regulations, service accessibility, housing standards, and recreational opportunities, integrating these elements into a cohesive legal framework. It should also ensure that HGMS receives adequate financial backing, including tax incentives and financial subsidies for housing facilities, bed availability, and operational costs, enhancing its sustainability and scalability (34, 35). Furthermore, optimizing the elder care industry’s structure will ensure high-quality resources meet increasing demand. In order to achieve this goal, efficient operational mechanisms should be established, such as public-private NGO coordination committees at the municipal level, including civil affairs departments, medical providers, community organizations, and HGMS resident representatives. Finally, HGMS initiators should consider outsourcing management tasks and leasing resources to professional organizations or intermediaries to boost the model’s sustainability. By adopting market-oriented operational models similar to those used by homestay platforms (36, 37), combined with cross-subsidy mechanisms, can further strengthen this approach. Under such frameworks, higher-income HGMS units subsidize affordable units, while initiators meeting affordability thresholds can benefit from tax credits, incentivizing balanced and inclusive development. HGMS can enhance its sustainability while reducing reliance on individual initiators, thereby increasing its vitality and longevity.

## Limitations and further studies

While this study provides a comprehensive analysis and valuable insights into the emerging HGMS older adult care model, it is crucial to acknowledge certain limitations. Firstly, the generalizability of the research may be affected by constraints such as budget, team size, and available resources. Consequently, the findings stem from specific geographic areas (Hangzhou) and particular HGMS types, which may limit the generalizability of findings to different regions or countries. Future research could prioritize expanding sample diversity across multiple dimensions: (1) Geographical diversity by comparing implementations in urban, suburban, and rural settings; (2) Socioeconomic inclusion, with a particular focus on low-income households and marginalized communities. Secondly, this study did not explore potential cultural or regional variations in the implementation and effectiveness of the HGMS model, presenting a significant area for further exploration. Understanding how these variations may impact the adoption and success of the HGMS model is crucial for its widespread implementation. Future investigations should incorporate cross-cultural validity testing of HGMS frameworks, with a particular focus on comparing Eastern collectivist societies and Western individualist contexts to examine how cultural differences influence the effectiveness and adaptability of these models. Finally, the study did not account for the potential impact of unforeseen events. Overall, the model seems somewhat fragile, as evidenced by the disruption caused by the COVID-19 pandemic.

Based on the study’s findings, several promising avenues for future research could further refine the HGMS model. First, subsequent studies should investigate the effectiveness of various HGMS models in addressing the diverse needs of older individuals, considering differing older adult care requirements and cultural contexts. Comparative studies across different regions, cultures, or countries could yield valuable insights into the factors that drive successful implementation. Additionally, exploring how community engagement and social participation can enhance well-being within HGMS environments is another important area for research. By addressing these gaps, future studies can play a pivotal role in advancing development of more effective and sustainable HGMS solutions tailored to urban populations.

## Conclusion

HGMS has played a pivotal role in the formulation of a sustainable strategy for older adult care, defined by the principles of “active aging and enjoyment in later life,” which not only alleviates the conventional supply-demand discrepancies associated with older adult care but also promotes the recognition of older adults’ intrinsic value. By facilitating resource sharing and harnessing the internal motivation of older individuals, HGMS has developed a self-organizing framework for mutual assistance, offering innovative solutions to the challenges posed by global aging.

Despite facing external challenges, including insufficient societal backing and regulatory frameworks, as well as internal barriers such as high entry thresholds and limited resources, the HGMS model represents a significant innovation that complements existing care systems. It signifies a fresh direction for supply-side structural reform in older adult services, aiming to elevate the well-being and happiness of seniors while fostering social harmony.

The HGMS model is founded on the principles of social inclusion and mutual assistance, establishing an organizational structure and implementation mechanism that effectively addresses the needs of the older adult population. This model promotes active aging through self-organization among older adults. Internal operations are facilitated by mutual assistance among members, while contractual norms contribute to the development of a supportive social environment. Furthermore, HGMS transitions older adult care services from the private domain of families to the public sphere, offering practical evidence for achieving health, well-being, and sustainable active aging within human society.

## Data Availability

The original contributions presented in the study are included in the article/supplementary material, further inquiries can be directed to the corresponding author.
